# The Healthiness of Food and Beverages on Price Promotion at Promotional Displays: A Cross-Sectional Audit of Australian Supermarkets

**DOI:** 10.3390/ijerph17239026

**Published:** 2020-12-03

**Authors:** Lily Grigsby-Duffy, Sally Schultz, Liliana Orellana, Ella Robinson, Adrian J. Cameron, Josephine Marshall, Kathryn Backholer, Gary Sacks

**Affiliations:** 1 Global Obesity Centre (GLOBE), Institute for Health Transformation, Deakin University, Geelong, VIC 3220, Australia; sally.schultz@deakin.edu.au (S.S.); ella.robinson@deakin.edu.au (E.R.); adrian.cameron@deakin.edu.au (A.J.C.); josephine.marshall@deakin.edu.au (J.M.); kathryn.backholer@deakin.edu.au (K.B.); gary.sacks@deakin.edu.au (G.S.); 2 Biostatistics Unit, Faculty of Health, Deakin University, Geelong, VIC 3220, Australia; l.orellana@deakin.edu.au

**Keywords:** price promotion, food policy, food environment, nutrition, health equity

## Abstract

Supermarket environments can strongly influence purchasing decisions. Price promotions are recognised as a particularly persuasive tactic, but the healthiness of price promotions in prominent in-store locations is understudied. This study compared the prevalence and magnitude of price promotions on healthy and unhealthy food and beverages (foods) displayed at prominent in-store locations within Australian supermarkets, including analyses by supermarket group and area-level socio-economic position. A cross-sectional in-store audit of price promotions on foods at key display areas was undertaken in 104 randomly selected stores from major Australian supermarket groups (Woolworths, Coles, Aldi and independents) in Victoria, Australia. Of the display space dedicated to foods with price promotions, three of the four supermarket groups had a greater proportion of display space devoted to unhealthy (compared to healthy) foods at each promotional location measured (end of aisles: 66%; island bins: 53%; checkouts: 88%). Aldi offered very few price promotions. Few measures varied by area-level socio-economic position. This study demonstrated that price promotions at prominent in-store locations in Australian supermarkets favoured unhealthy foods. Marketing of this nature is likely to encourage the purchase of unhealthy foods, highlighting the need for retailers and policy-makers to consider addressing in-store pricing and placement strategies to encourage healthier food environments.

## 1. Introduction

Globally, excess body weight and unhealthy diets are leading risk factors for death and disability [1]. Furthermore, a socio-economic gradient exists, whereby more disadvantaged socio-economic groups have disproportionately lower-quality diets and a greater burden of diet-related disease [2,3,4,5].

Food environments, of which supermarkets are a significant component, are an important determinant of dietary behaviour [6]. Australia’s supermarket industry is highly concentrated, with two supermarket groups (Woolworths and Coles) holding the majority of the grocery market share (67.5%) [7].

The marketing techniques used in supermarkets can strongly influence consumers’ purchasing decisions [8,9], and are often designed to encourage unplanned or impulse purchases [10]. Price discounting can be particularly effective at influencing purchasing decisions [11,12,13], and may be an important driver for those on lower incomes who tend to be more price sensitive [14]. Moreover, research suggests that products purchased on price promotion are consumed at a faster rate than usual due to the perceived cost of purchasing or replacing the product being low and the salience of the product being high (e.g., occupying additional shelf space at home) [15]. 

Australia has typically been dominated by grocery retailers that practice high–low pricing strategies [16] (i.e., price promotions). The proportion of grocery sales purchased from price promotions in Australia is one of the highest in the world [17]. Competition from discount stores is low in Australia, with the exception of Aldi which arrived in 2001 [18].

Price promotions are often used in conjunction with other in-store marketing strategies to increase salience and further encourage sales [19]. For example, there is evidence to suggest that price-promoted products that are promoted in combination with a placement strategy (e.g., a dedicated display) are more effective at increasing the demand for the product compared to price promotions alone [19]. The promotion of food and beverages using price discounts and placement strategies in store is likely to impact purchasing behaviour and ultimately population eating behaviour. It is therefore important to understand the degree to which these marketing tactics are applied to healthier and less-healthy products.

Previous studies have observed an over-representation of price promotions on unhealthy (compared to healthier) products in supermarkets in Australia, New Zealand, The Netherlands, US, Canada, and the Republic of Ireland [20,21,22,23,24,25,26,27]. Most of these studies assessed supermarket circulars [24,25] or online data [22,23] and, therefore, were unable to investigate the interaction between price promotions and placement in store, nor how findings may vary across stores located in areas with different levels of socio-economic disadvantage. 

Of the few in-store audits of food and beverage price promotions, two studies have considered positioning. An audit of breakfast cereals in five Canadian stores assessed multiple components of the food environment separately, including price promotions, placement and positioning strategies, finding that each tactic was biased towards unhealthy cereals [27]. A recent study conducted in the Republic of Ireland [26] assessed in-store price promotions by food category and for descriptive purposes also recorded whether the product was in a ‘promotional area’ (end-of-aisle displays or island bins). However, neither study considered differences by socio-economic position (SEP) [26,27]. Two in-store audits explored differences by SEP [20,21]. One study conducted in New Zealand in 2007 categorised SEP by supermarket format (full-service supermarkets vs. discount supermarkets) [20], whilst the other study conducted in the US between 2010 and 2012 assessed community-level SEP based on median household income [21]. Neither study found any significant differences in the prevalence of food and beverage price promotions across SEP levels.

This is the first study assessing the overall healthiness of food and beverage price promotions at key in-store areas and exploring the differences by area-level socio-economic disadvantage. In this study, we aimed to compare the prevalence and magnitude of price promotions on different categories (healthy and unhealthy) of food and beverages (foods) displayed at prominent in-store locations within Australian supermarkets, including analyses by supermarket groups and area-level SEP.

## 2. Materials and Methods 

### 2.1. Study Design and Sampling

A cross-sectional study of the three largest supermarket retailers in Australia (grocery market shares 2017–2018: Woolworths 37.2%; Coles 30.3%; Aldi 9.2% [7]) and independently owned supermarket (referred to as ‘Independents’) stores was conducted. Independents comprised mostly of Independent Grocers of Australia (IGA) (Canning Vale, Australia) stores (Metcash (Macquarie Park, Australia), owners of IGA and other independents, have 7.4% of the market share) [7]. These supermarket groups represent the majority of the grocery market shares in Australia.

Stores from the four supermarket groups (Woolworths (Bella Vista, Australia), Coles (Hawthorn East, Australia), Aldi (Essen, Germany), Independents) were sampled from Statistical Area Level 2 (SA2) areas in ‘major cities of Victoria’, of which there are 309, as categorised by the Australian Bureau of Statistics (ABS) [28]. SA2s (average population 10,000) are characterised as “communities that interact together socially and economically” [28]. Each SA2 in Victoria was categorised into quintiles of the Socio-Economic Indexes for Areas (SEIFA) Index of Relative Socio-economic Advantage and Disadvantage—an index developed by the ABS that ranks areas according to relative socio-economic advantage and disadvantage using twenty variables related to income, employment, family composition, housing benefits, car ownership, ethnicity, English language proficiency and residential overcrowding [29]. The SEIFA quintiles range from quintile 1 (Q1: most disadvantaged) to quintile 5 (Q5: least disadvantaged). A random sequence of SA2s for each SEIFA quintile was produced from which SA2 areas were randomly selected, stratified by SEIFA quintile.

Within each of the randomly selected SA2 areas, a list of stores was identified on Google Maps. One store from each of the four supermarket groups was selected from each SA2. If there was more than one store per supermarket group in the SA2, one of the stores was randomly selected. If a supermarket group did not have a store located in the SA2, a store from that supermarket group was selected from the next SA2 on the list that was in the same SEIFA quintile. This sampling process continued until 104 stores meeting the quota (26 stores from each supermarket group with ten stores from SEIFA Q1 and sixteen stores from SEIFA Q2–Q5, four from each quintile) were selected. In total, 57 SA2′s were included in the final sample. As lower-SEP groups have lower-quality diets and more diet-related disease [2,3,4,5], the most disadvantaged SEIFA quintile (Q1) was oversampled (Q1: *n* = 40; Q2–Q5: *n* = 16 each) in order to increase the power to detect differences between stores from this quintile and the other quintiles combined (Q2–Q5).

A sample size of 104 stores was selected based on feasibility as no previous studies have used comparable measures to the ones in this study. The sample size selected (*n* = 104) encompasses a large proportion (18%) of all full-service supermarkets (excluding express supermarkets) located in the ‘major cities of Victoria’.

### 2.2. Audit Tool

An audit tool (Supplementary File S1) was developed to assess the healthiness of the supermarket food environment as part of a larger project monitoring supermarket environments in Australia (reported elsewhere) [30]. The tool was based on the validated INFORMAS tool [31] previously used in New Zealand. Price promotion measures, which were previously not included in the tool, were added.

Data were collected on all foods placed in the following prominent displays in each store: (1) end of aisles; (2) checkouts; and (3) island bins (temporary, non-fixed displays) (see Supplementary File S1 for picture examples of the display types). At each display, the following information was recorded: (1) location (end-of-aisle displays at the front or back of the store; staffed- or self-checkout displays; island bins near store entrance, near staffed- or self-checkouts, near end of aisles, or elsewhere); (2) display size (island-bin surface area [m^2^]; end of aisle component defined as left or right side, or centre; checkout end [located at the beginning of the checkout facing in towards the store] or side [located above the conveyer belt]); (3) product categories on display, including non-food; (4) estimated percentage of space taken up by each product category (e.g., a product occupying one shelf on a five-shelf display would equate to 20% of the display space); and (5) price promotion information. Price promotion information included whether the product was price promoted (yes/no), the type of price promotion strategy (temporary or permanent), and the magnitude of discount on temporary promotions (percentage off recommended retail price, for multi-buys, this was calculated as the percentage difference between the cost of the multiple items total sale price vs. the total cost of buying the same number of items at their regular retail price). Temporary price promotions were those that applied for a limited time and included discounts (where an item is reduced against a reference price, e.g., “50% off”), multi-buys (requires the purchase of multiple products to receive the discount, e.g., “buy one get one free”), and discounts on products with no regular retail price (e.g., “fresh specials” on fruit and vegetables). Permanent price promotions were defined as those promoted through a price reduction signal (e.g., “everyday low price”) and did not have an immediate time limit.

To minimise the disruption to customers in store, data collection in checkout areas regarding ‘type of price promotion strategy’ was limited to whether price promotions were temporary or permanent and magnitude of discount was not collected.

### 2.3. Data Collection

Data were collected in store, on paper, by one of two auditors trained in the use of the audit tool. Data were collected across 13 weeks, from May to July 2019. This period was selected to avoid major holidays, such as Easter.

Data collection across SEIFA quintiles and supermarket groups were spread across the data collection period and across auditors in an attempt to reduce systematic errors. Inter-rater reliability was assessed by the two auditors assessing four of the same stores (one from each supermarket group) on the same day. All price promotion measures showed high inter-rater reliability (intra-class correlation coefficients = 0.951 to 0.999).

### 2.4. Data Management

Data were entered, coded and cleaned using Microsoft Excel.

The Australian Dietary Guidelines (ADGs) [32] were used as the basis for categorising each product as ‘healthy’ or ‘unhealthy’, and additional categories were assigned for ‘non-food’ (e.g., toiletries, supplements, and infant formula) or ‘unable to categorise’ (e.g., some mixed foods). Items were categorised as healthy if they were one of the ADGs recommended ‘Five Food Groups’ (vegetables/legumes; fruit; grain foods; meat/eggs/tofu/nuts/seeds/legumes; milk/yoghurt/cheese/alternatives), unsaturated spreads and oils or water. Items were categorised as unhealthy if they were discretionary products according to the ABS Discretionary Food List (e.g., chocolate, chips, soft drink) [33]. Discretionary foods and beverages are not nutritionally required as part of a healthy diet and are typically ‘energy-dense’ but ‘nutrient-poor’ [32]. Categorisation was cross-checked by a dietitian.

The proportion of display space allocated to each product was calculated as the estimated proportion of space taken up by the product in the display multiplied by a weight reflecting display size. End-of-aisle displays: the centre display was weighted by 0.5, and the side displays by 0.25 each. Island bins: small bins (<1 m^2^ surface area) were weighted by 0.5, medium bins (1–1.5 m^2^) by 1, large bins (1.5–2 m^2^) by 1.5, and extra-large bins (>2 m^2^) by 2. Checkouts: side and end displays were considered comparable and therefore weighted by 1. The proportion of the total display space devoted to foods is used throughout and referred to as food display space.

The following derived measures were calculated: (1) the proportion of food display space with products on price promotion; (2) the proportion of food display space with products on price promotion categorised as unhealthy; and (3) the mean magnitude of discount for food overall, for categories of healthiness and the difference between healthy and unhealthy foods.

Independent variables included supermarket group (Woolworths, Coles, Aldi, Independents) and SEP (SEIFA quintiles categorised into two groups: Q1 (most disadvantaged) and Q2–Q5).

### 2.5. Data Analysis

Data were analysed in STATA SE 15 (StataCorp, College Station, TX, USA). For each measure at each prominent location (end of aisle, checkouts, island bins altogether and for each location), a linear model was fitted including supermarket group (Woolworths, Coles, Aldi, Independents), SEP (SEIFA Q1, Q2–Q5) and the interaction of supermarket group and SEP. Pairwise comparisons for differences between supermarket groups were Sidak adjusted. For descriptive purposes, we calculated the ratio between the proportions of food display space with price promoted foods dedicated to unhealthy versus healthy products.

### 2.6. Ethical Approval

Ethics approval was granted by Deakin University Human Ethics Advisory Group (HEAG-H 57_2019). Written permission from the head office of three supermarket groups (Woolworths, Coles, Aldi) was obtained. As Independents have no single head office, informed consent was sought on arrival at the store.

## 3. Results

One hundred and four stores were audited. For each supermarket group, ten stores were sampled from SEIFA Q1 and sixteen stores from SEIFA Q2–Q5.

At Aldi, minimal food display space was dedicated to price promotions at end-of-aisle (3.4%) or checkout displays (0.3%). Therefore, Aldi was excluded from the linear models and not considered in the analysis for end of aisles or checkouts.

Table 1 describes the characteristics of the foods on price promotion. For all supermarket groups and prominent in-store locations, the predominant type of price promotion was temporary, as opposed to permanent, price reductions (end of aisles: 85.7%; island bins: 86.7%; checkouts: 79.1%). At end-of-aisle and island-bin displays, the most common type of temporary discount was a discounted price, whilst multi-buys were the least common at island bins (9%) and fresh specials were the least common at end of aisles (2.3%). Details of the type of discount was not recorded at checkouts.

### 3.1. Proportion of Food Display Space with Products on Price Promotion in Prominent Locations

The mean proportion of food display space dedicated to price promotions at each in-store supermarket location is described below and summarised in Table 2. Interactions between supermarket group and SEP are described below and significant interactions are indicated by an asterisk in Table 2.

#### 3.1.1. End of Aisles

At end-of-aisle displays, the mean percentage of food display space occupied by products on price promotion varied by supermarket group (Table 2). Woolworths (91.1%) and Coles (83.7%) had significantly more food display space devoted to price-promoted products than Independents (46.1%) (both comparisons *p* < 0.001).

The interaction between supermarket group and SEP for the proportion of food display space occupied by products on price promotion was significant at end of aisles (*p* = 0.004). Percentages for Woolworths and Coles were similar across stores in Q1 and Q2–Q5, but Independents had a higher proportion of food display space occupied by price-promoted products in stores from Q1 compared to Q2–Q5 (mean difference = 32.7% [95% CI: 11.3, 54.2], *p* < 0.001).

#### 3.1.2. Island Bins

At island bins, Woolworths (71.6%) and Coles (63.2%) had significantly more food display space devoted to price-promoted products compared to Independents (34.4%) (both comparisons *p* < 0.001) and Aldi (26.7%, both *p* < 0.001) (Table 2).

There was a significant interaction between supermarket group and SEP for island-bin space devoted to price promotions (*p* = 0.04). This interaction is reflected in the fact that Woolworths and Aldi had a lower proportion of display space devoted to price promotions in Q1 compared to Q2–Q5 (2.2% lower and 8.9% lower, respectively), while Coles and Independents had a larger proportion of display space devoted to price promotions in Q1 compared to Q2–Q5 (8.5% larger and 17.9% larger, respectively).

#### 3.1.3. Checkouts

At checkouts, the mean proportion of food display space occupied by price promotions was significantly greater in Woolworths (61.7%) and Coles (68.6%) compared to Independents (16.0%) (both comparisons *p* < 0.001) (Table 2).

For the proportion of checkout display space occupied by products on price promotion, there was no significant interaction between supermarket group and SEP, nor was there a significant difference between Q1 and Q2–Q5 overall or by supermarket group.

### 3.2. Proportion of Price-Promoted Food and Beverage Display Space Dedicated to Unhealthy Products in Prominent Locations

**Figure 1:**
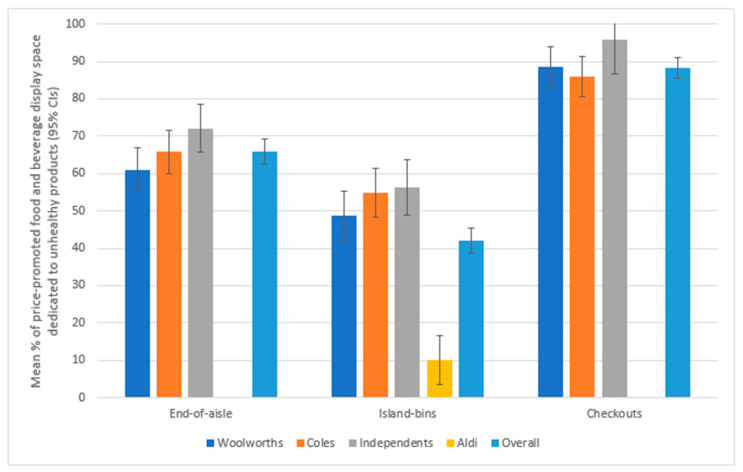
Mean proportion (%) of price-promoted food and beverage display space dedicated to unhealthy products^†^ at each in-store supermarket location, by supermarket group in 2019. Figure depicts results of linear models that included supermarket group, SEP and interaction supermarket group by SEP. Aldi was not included in the linear model or overall mean % for end-of-aisle or checkout displays due to there being insufficient numbers of price promotions in those location. † Foods were classified as healthy based on the Australian Bureau of Statistics Discretionary Food List.

The mean proportion of price-promoted food and beverage display space dedicated to unhealthy product is described below and summarised in Table 2 and presented in Figure 1.

#### 3.2.1. End of Aisles

Of the foods on price promotion at end-of-aisle displays, the three products with the largest proportion of display space were unhealthy beverages (which included soft drinks, energy drinks, sports drinks and iced tea, 15.0% 95% CI: 11.9, 18.1), confectionary (14.3% 95% CI: 11.1, 17.5), and crisps (9.3% 95% CI: 6.6, 12.1).

Of the foods on price promotion, there was twice as much display space at end of aisles devoted to unhealthy (65.9%) compared to healthy (33.2%) products across Woolworths, Coles and independents (Table 2). Independents had the highest proportion of unhealthy price-promoted products, followed by Coles then Woolworths (72.1%, 65.8%, and 61.0%, respectively). The difference between Independents and Woolworths was significant (*p* = 0.045).

There was no significant interaction between supermarket group and SEP or differences between SEP levels within supermarket group in the proportion of price-promoted display devoted to unhealthy price promotions.

#### 3.2.2. Island Bins

Of the foods on price promotion at island-bin displays, the three products with the largest proportion of display space were fruits and vegetables (34.3% 95% CI: 27.8, 40.7), unhealthy beverages (11.7% 95% CI: 8.9, 14.5), and confectionary (11.4% 95% CI: 9.4, 13.4).

Aldi (10.1%) had significantly less display space in island bins with unhealthy price-promoted products compared to Woolworths (48.8%), Coles (54.9%) and Independents (56.2%), all comparisons *p* < 0.001 (Table 2). For island bins, there was no significant interaction between supermarket group and SEP in the proportion of price-promoted food and beverage display space devoted to unhealthy products. Nor were there any significant differences in this measure between SEP levels overall or by supermarket group.

The proportion of price-promoted food and beverage display space devoted to unhealthy products varied by the location of island-bin displays. Table 3 presents the mean proportion of price-promoted display space dedicated to unhealthy products by island bin location (i.e., those located around the checkouts, near end of aisles, near the entrance of the store, and elsewhere in store). Island bins near checkouts and end of aisles had the most price-promoted food and beverage display space devoted to unhealthy products (77.8% and 72.2%, respectively). Island bins elsewhere in store and near the entrance had the least price-promoted food and beverage display space dedicated to unhealthy products (41.3% and 23.5%, respectively). Furthermore, there was a significantly larger proportion of price-promoted food and beverage display space devoted to unhealthy products in stores from Q1 compared to Q2–Q5 for island bins near checkouts (mean difference = 14.1% [95% CI: 2.5, 25.7], *p* = 0.02) and island bins elsewhere in store (mean difference = 15.6% [95% CI: 4.5, 26.8), *p* = 0.007). There was no significant interaction between supermarket group and SEP or differences between SEP levels within supermarket groups for either of these measures.

#### 3.2.3. Checkouts

Of the foods on price promotion at checkout displays, the three products with the largest proportion of display space were confectionary (69.8% 95% CI: 63.2, 76.3), unhealthy beverages (16.0% 95% CI: 11.4, 20.7), and healthier beverages (which included water, flavoured milk drinks and fruit juice) (14.7% 95% CI: 11.3, 18.1).

Of the price-promoted foods at checkouts, 88.3% of the display space was dedicated to unhealthy products across Woolworths, Coles and Independents (Table 2). For the proportion of price-promoted food and beverage display space devoted to unhealthy products, the interaction between supermarket group and SEP was significant (*p* = 0.03). The proportion of price-promoted food and beverage display space devoted to unhealthy products was greater in stores from Q1 compared to Q2–Q5 for Coles (difference of 11.1%), whilst Independents had a lower proportion in Q1 compared to Q2–Q5 (difference of 10.7%). No variation by SEP was observed in the proportion of price-promoted food and beverage display space devoted to unhealthy products at checkouts for Woolworths (difference < 1%).

### 3.3. Magnitude of Discount

**Figure 2:**
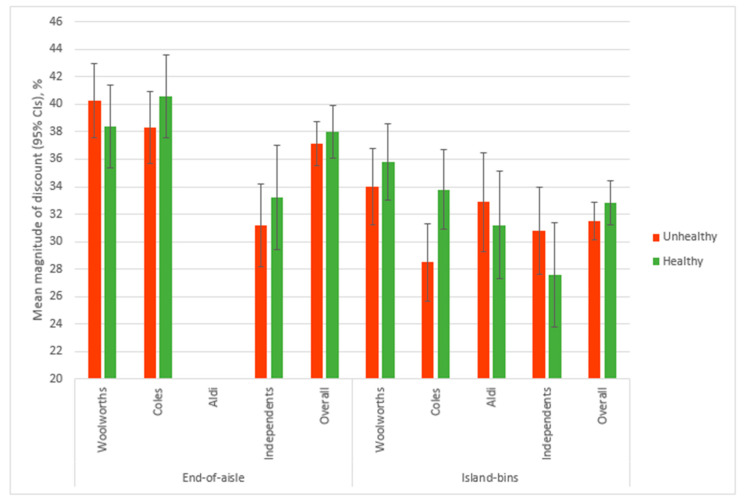
Mean magnitude of discount on food and beverage price promotions classified as healthy and unhealthy^†^ at prominent in-store supermarket locations, by supermarket group in 2019. Figure depicts results of linear models that included supermarket group, SEP and interaction supermarket group by SEP. Aldi was not included in the linear model or overall mean % for end-of-aisle due to there being insufficient numbers of price promotions in those location. † Foods were classified as healthy or unhealthy based on the Australian Dietary Guidelines and the Australian Bureau of Statistics Discretionary Food List.

The mean magnitude of discount was calculated for products on temporary price promotions at end-of-aisle and island-bin displays. Permanent price promotions and specials where there was no regular retail price (e.g., fresh fruit and vegetables), were not included. Table 4 shows the mean magnitude of discount by supermarket group and SEP. Figure 2 shows the mean magnitude of discount on food and beverage price promotions classified as healthy and unhealthy at prominent in-store supermarket locations, by supermarket group.

#### 3.3.1. End of Aisles

At end of aisles, the overall mean magnitude of discount for foods was 36.7% (Table 4). The mean magnitude of discount for both unhealthy and healthy products located at end of aisles was significantly higher in Woolworths (unhealthy: 40.3%; healthy: 38.4%) and Coles (unhealthy: 38.3%; healthy: 40.6%) compared to Independents (unhealthy: 31.2%; healthy: 33.2%, both comparisons *p* < 0.001). There was no significant interactions between supermarket group and SEP in the magnitude of discount at end-of-aisle displays. There were no significant differences overall, by supermarket group or by SEP in the mean difference between the magnitude of discount for healthy and unhealthy products.

#### 3.3.2. Island Bins

The overall mean magnitude of discount was 31.5% at island bins (Table 4). The mean magnitude of discount was significantly higher in Woolworths compared to Independents overall (34.7% vs. 29.0%, *p* = 0.045) and for healthy products (35.8% vs. 27.6%, *p* = 0.005) but was not different for unhealthy products (Woolworths: 34.0%; Independents: 30.8%).

Within supermarket groups, Coles discounts were on average 5.2% greater for healthy products (compared to unhealthy), whilst Independents had a 2.9% greater discount on unhealthy products (compared to healthy). There was no significant interactions between supermarket group and SEP in the magnitude of discount at island-bin displays or differences between SEP levels overall or within supermarket groups.

## 4. Discussion

This study assessed price promotions at prominent in-store locations in supermarkets in Victoria, Australia. Among the supermarket groups studied, Aldi consistently had the least display space dedicated to price promotions. At each of the promotional locations measured, the proportion of display space dedicated to foods with price promotions was greatest in Woolworths and Coles compared to Independents. Of the display space occupied by foods with price promotions, Woolworths, Coles and Independents all had a greater proportion of space devoted to unhealthy (compared to healthy) foods. Across these three supermarket groups, of the display space dedicated to price promotions, 88% at checkouts, and 66% at end of aisles was for unhealthy foods. The magnitude of discount applied to price promotions in-store was similar for unhealthy and healthy foods, with some variation seen by supermarket group. At most of the in-store locations, measures were similar across stores located in the most disadvantaged areas and stores in other areas. However, some important differences by area-level SEP were observed. The findings from this study suggest that, overall, there is greater exposure to unhealthy price promotions compared to healthy price promotions at prominent locations in store; however, a person’s exposure to price promotions can vary depending on the area and supermarket group at which they shop.

The finding that price promotions are applied more frequently to unhealthy compared to healthy products is similar to findings from previous studies [20,21,22,23,24,25,26,27]. For example, a recent study of food prices in the online store of Australia’s largest supermarket group found that unhealthy food was price promoted almost twice as often as healthy food [23]. The unique contribution of this study is a demonstration that this bias towards unhealthy products is even more pronounced for foods in prominent locations in store, particularly at checkouts where 88% (across Woolworths, Coles, and Independents) of price promotion display space was for unhealthy products. As the vast majority of Woolworths and Coles supermarket sales are in store (rather than online) [34,35], and the placement of products can enhance the effect of price promotions [19], the promotion of unhealthy foods at prominent locations observed in this study is likely to have a substantial impact on Australians purchasing decisions and population diets.

In this study of products in prominent locations only, there was little difference in the magnitude of discount between healthy and unhealthy products. This is similar to the findings from another in-store audit conducted in the Republic of Ireland that found no differences in the magnitude of discount by food categories [26]. However, it is inconsistent with a previous study of online data from Australian supermarkets that found the mean weekly magnitude of discount over the year was greater for unhealthy compared to healthy foods (25.9% vs. 15.4%) [23]. The differences in the results of the current study to those of the earlier Australian study of online data likely reflect differences in the magnitude of discounts offered on products placed at prominent in-store locations compared to elsewhere in store (and online), rather than changes in supermarket practices over time. In this study, usual price points were not collected. There is a growing body of research from Australia that suggests healthy diets are cheaper than unhealthy diets [36,37,38]. However, these studies do not take into account price promotions.

In this study, few differences by area-level SEP were observed. This is partially consistent with two previous studies conducted in New Zealand (2007) [20] and the US (2010-2012) [21] that assessed in-store price promotions and found no differences by SEP. While it would appear as though, for the most part, the major Australian supermarket groups apply their price promotion strategies in similar ways across stores located in different SEP areas, it is nevertheless important to reflect on potential differences in the impact of price promotions on different SEP groups. It is well established that the perceived price and affordability of food is an important determinant of food choices, particularly for those in lower SEP populations [14,39,40]. Therefore, pricing strategies that are biased towards unhealthy products are likely to disproportionality affect lower SEP populations, although this needs further investigation

To our knowledge, this is the first study to assess the overall healthiness of food and beverage price promotions at key in-store areas and explore the differences by area-level socio-economic disadvantage. It is also the first in-store audit of food and beverage price promotions conducted in Australia. Other strengths of this study include the large sample of 104 stores and that stores were randomly selected.

Limitations should be considered when interpreting the results of this study. Firstly, due to the limited study duration (13 weeks) seasonal variation in price promotions could not be assessed. However, data from a recent online assessment of supermarket price promotions in Australia found that there was little seasonal variation when looking at price promotions in the store [23].

Secondly, this study also has limited generalisability as, due to resource constraints, only major cities in Victoria were sampled. In the testing phase of this study, a small sample of stores (*n* = 12) in Brisbane, Australia were audited. The findings from Brisbane stores were similar to those reported in this study, suggesting that the way that supermarkets use price promotions at prominent display sites may be comparable in other cities in Australia, although this needs further examination. Rural areas have disproportionately poorer health compared to urban areas [41] and, therefore, rural food environments are an important area of future study.

Furthermore, this study considers only one aspect of the food environment. However, the healthiness of price promotions in this study and differences by supermarket group is reflective of the other marketing strategies (shelf-space devoted to healthy vs unhealthy foods and proportion of prominent displays free from unhealthy foods) that this research team audited as part of a separate report [30]. Likewise, there are other factors that can influence consumer purchasing behaviour (e.g., nutritional knowledge) that have not been explored in this study. Dietary behaviour is influenced by a broad array of factors ranging from an individual’s knowledge and attitudes to structural aspects of the environment [42].The findings from this study have important implications for public health and policy. With Woolworths, Coles, and Metcash (IGA) combined representing 75% of the grocery market share in Australia [7], the high percentage of unhealthy food on price promotions at prominent displays likely reflects the lived experience for the majority of Australians when shopping in supermarkets. Research has found that, independently, price promotions and placement strategies can increase food and beverage purchases and consumption [15,43,44], and combined, placement strategies can exacerbate the promotional effect of price promotions [19]. It is therefore reasonable to assume that an environment that favours price promotions on unhealthy foods and beverages, especially when located in promotional areas, will result in increased sales of unhealthy products. Accordingly, reducing the promotion of unhealthy foods and beverages through price promotions in supermarkets may help to improve population diets, and could contribute to reducing the socio-economic gradient in health, although further research is needed in this area.

A comprehensive meta-analysis of nudge based interventions in food retail settings supports the idea that in-store strategies can result in healthier choices [45]. The meta-analysis found that convenience nudges (i.e., nudges that make it physically easier to select healthier options) were effective at improving the healthiness of food and beverage choices. Additionally, a recent randomised controlled trial conducted in remote Australian stores implemented an intervention (The Healthy Stores 2020 strategy) to restrict the marketing of unhealthy food and beverages [46]. The intervention combined seven strategies including the restriction of price promotions and removal of unhealthy products from key promotional locations. The Healthy Stores 2020 strategy resulted in a significant reduction in sales of free sugar [46]. Furthermore, a modelling study estimated that a policy restricting temporary price promotions on sugar sweetened beverages in Australian food retail settings was likely to reduce mean population per capita daily sugar intake and mean population body weight [47]. Future empirical research should assess the effect of food and beverage price promotion interventions on consumer behaviour and health outcomes.

Whilst retailers and manufacturers may benefit from the short-term increase in sales gained from offering price promotions [12,13], there is also the risk that retailers and manufacturers can become trapped in a ‘price promotion spiral’ [13]. Repeated exposure to price promotions can increase customers’ price sensitivity. In turn, highly price sensitive customers result in retailers needing to offer even more frequent and/or greater magnitude of discount [13]. This situation has been evident in Australia with the two leading supermarkets (Coles and Woolworths), labelled as being in a “price war”, continuously offering a high number of price promotions [18]. Australians were also found to be the most price sensitive grocery shoppers compared to customers from 35 other countries [48]. Further research is needed in order to understand how any action to reduce unhealthy food and beverage price promotions would impact dietary choices and be perceived by the public and other key stakeholders.

Due to competitive pressures, voluntary changes in current discounting cycles are perhaps unlikely. Accordingly, government regulation may play an important role in creating standards for food retail marketing practices. Policies addressing the relative price and affordability of healthy foods have been recognised as a key strategy to improve diet [49,50,51,52,53]. Recently, the UK Government released a new obesity strategy which included restrictions on volume-based price promotions on unhealthy foods [54], making them the first country to implement a policy on restricting food and beverage price promotions as a strategy to improve population health.

## 5. Conclusions

This study assessed food and beverage price promotions at prominent in-store locations in supermarkets in Victoria, Australia. This study demonstrated that the proportion of price promotion display space was greater for unhealthy food and beverages than healthier products at end-of-aisle and checkout displays; however, exposure to price promotions at prominent areas can vary depending on area-level SEP and supermarket group. Marketing (through price promotions and placement strategies) that is biased towards unhealthy foods and beverages is likely to encourage the purchase of unhealthy foods, highlighting the need for action by both supermarket retailers and policy-makers to restrict both the placement and price promotion of unhealthy products in food retail settings.

## Figures and Tables

**Table 1:** Characteristics of food and beverage price promotions at prominent in-store supermarket locations, by supermarket group.

	End of Aisle							Island Bins ^3^						Checkouts			
Supermarket Group	Mean % of Display Space Devoted to Food and Beverages (SD)	Mean % of Food and Beverage Display Space Price Promoted (SD)	Mean % of Price Promotions That Were Temporary ^1^ (SD)				Mean % of Price Promotions That Were Permanent ^2^ (SD)	Mean % of Food and Beverage Display Space Price Promoted (SD)	Mean % of Price Promotions That Were Temporary (SD)				Mean % of Price Promotions That Were Permanent (SD)	Mean % of Display Space Devoted to Food and Beverages (SD)	Mean % of Food and Beverage Display Space Price Promoted (SD)	Mean % of Price Promotions That Were Temporary (SD)	Mean % of Price Promotions That Were Permanent (SD)
			All	DISCOUNTS	Multi-Buys	Fresh Specials			All	Discounts	Multi-Buys	Fresh Special					
Woolworths	58.2 (1.2)	91.1 (1.3)	82.6 (1.5)	78.7 (1.5)	0.5 (2.4)	3.4 (0.5)	17.4 (1.5)	71.6 (1.6)	83.4 (1.5)	60.6 (1.9)	3.3 (0.4)	19.5 (1.5)	16.6 (1.5)	46.2 (2.3)	61.7 (3.5)	73.8 (3.9)	26.2 (3.9)
Coles	63.9 (1.3)	83.7 (1.3)	87.0 (2.0)	77.4 (2.4)	9.2 (1.2)	0.3 (0.3)	13.0 (2.0)	63.2 (2.7)	76.0 (2.1)	39.5 (2.6)	20.0 (1.3)	16.4 (1.9)	24.0 (2.1)	52.4 (3.4)	68.6 (4.0)	79.3 (3.8)	20.7 (3.8)
ALDI	64.8 (2.3)	3.4 (1.8)	100 (0)	90.7 (9.3)	0	9.3 (9.3)	0	26.7 (2.2)	100 (0)	23.3 (6.3)	0	76.7 (6.3)	0	54.9 (2.8)	0.3 (0.3)	100 (0)	0
Independents	58.1 (4.7)	46.1 (6.4)	81.2 (7.3)	69.4 (8.7)	11.9 (5.6)	0	18.8 (7.3)	34.4 (5.8)	88.3 (5.7)	66.5 (7.7)	13.4 (4.9)	8.5 (3.0)	11.7 (5.7)	65.0 (4.8)	16.0 (4.8)	90.0 (10.0)	10.0 (10.0)
Overall	61.3 (1.4)	56.1 (3.8)	85.7 (2.1)	77.4 (2.7)	6.0 (1.6)	2.3 (1.1)	14.3 (2.1)	49.0 (2.5)	86.7 (1.6)	46.6 (2.9)	9.0 (1.3)	31.1 (3.3)	13.3 (1.6)	54.6 (1.8)	36.6 (3.4)	79.1 (2.8)	20.9 (2.8)

^1^ Temporary price promotions apply for a limited time and include discounts (where an item is reduced against a reference price such as a previous, future, external, or recommended retail price, e.g., “50% off”), multi-buys (requires the purchase of multiple products to receive the discount, e.g., “buy one get one free”), and Fresh specials (discounts on fruit and vegetables with no regular retail price). ^2^ Permanent price promotions do not have an immediate time limit but are promoted through a price reduction signal, e.g., everyday low price. SD: standard deviation. ^3^ Mean % of display space devoted to food and beverages not reported for island bins as data were only collected on food and beverage island bins.

**Table 2:** Mean proportion (%) of food and beverage display space dedicated to price promotions, and mean proportion (%) of price-promoted food and beverage display space dedicated to unhealthy products at prominent in-store supermarket locations, by supermarket group and area-level socio-economic position.

		End of Aisle ^1^		Island Bins		Checkouts ^1^	
Supermarket Group, SEP	No. of Stores	Mean % of Food and Beverage Display Space Price Promoted (95% CIs)	Mean % of Price-Promoted Food and Beverage Display Space Dedicated to Unhealthy Products (95% CIs)	Mean % of Food and Beverage Display Space Price Promoted (95% CIs)	Mean % of Price-Promoted Food and Beverage Display Space Dedicated to Unhealthy Products (95% CIs)	Mean % of Food and Beverage Display Space Price Promoted (95% CIs)	Mean % of Price-Promoted Food and Beverage Display Space Dedicated to Unhealthy Products (95% CIs)
Woolworths	26	91.1 (82.7, 99.5)	61.0 (55.2, 66.8)	71.6 (64.8, 78.3)	48.8 (42.3, 55.3)	61.7 (53.3, 70.0)	88.5 (83.1, 94.0)
Q1	10	93.1 (82.1, 100)	62.3 (52.9, 71.6)	70.2 (59.3, 81.0)	50.9 (40.5, 61.4)	56.7 (43.2, 70.2)	88.2 (81.0, 95.3)
Q2–Q5	16	89.8 (81.1, 98.6)	60.2 (52.8, 67.6)	72.4 (63.8, 81.0)	47.5 (39.2, 55.8)	64.8 (54.2, 75.4)	88.8 (83.1, 94.4)
Coles	26	83.7 (75.3, 92.2)	65.8 (60.0, 71.6)	63.2 (56.4, 69.9)	54.9 (48.5, 61.4)	68.6 (60.2, 76.9)	85.9 (80.5, 91.3)
Q1	10	84.7 (73.6, 95.8)	62.0 (52.7, 71.3)	68.4 (57.5, 79.2)	57.3 (46.8, 67.7)	68.3 (54.9, 81.8)	92.5 (85.4, 99.7)
Q2–Q5	16	83.1 (74.4, 91.9)	68.2 (60.9, 75.6)	59.9 (51.4, 68.5)	53.5 (45.2, 61.7)	68.7 (58.1, 79.4)	81.4 (75.8, 87.1)
Aldi	26	n/a	n/a	26.7 (19.9, 33.4)	10.1 (3.5, 16.8)	n/a	n/a
Q1	10	n/a	n/a	21.2 (10.3, 32.0)	14.2 (3.2, 25.3)	n/a	n/a
Q2–Q5	16	n/a	n/a	30.1 (21.5, 38.7)	7.8 (0, 16.1)	n/a	n/a
Independents	26	46.1 (37.7, 54.5)	72.1 (65.7, 78.6)	34.4 (27.7, 41.2)	56.2 (48.8, 63.6)	16.0 (7.6, 24.3)	95.7 (86.7, 100)
Q1	10	66.2 (55.1, 77.3)	73.9 (64.5, 83.2)	45.4 (34.6, 56.3)	55.3 (44.3, 66.3)	19.1 (5.6, 32.5)	89.3 (79.2, 99.4)
Q2–Q5	16	33.5 (24.7, 42.3)	70.5 (61.6, 79.4)	27.5 (19.0, 36.1)	57.0 (47.0, 67.0)	14.1 (3.4, 24.7)	100 (89.9, 100)
Overall	104	73.6 (69.7, 77.6) *	65.9 (62.5, 69.4)	49.0 (45.6, 52.3) *	42.0 (38.7, 45.4)	48.7 (43.9, 53.6)	88.3 (85.5, 91.2) *
Q1	40	81.4 (74.0, 88.7)	66.0 (60.6, 71.4)	51.3 (45.9, 56.7)	44.9 (39.6, 50.3)	48.0 (40.3, 55.8)	90. 2 (85.0, 95.4)
Q2–Q5	64	68.8 (63.0, 74.6)	65.8 (61.3, 70.3)	47.5 (43.2, 51.8)	40.1 (35.8, 44.4)	49.2 (43.1, 55.3)	87.5 (83.2, 91.8)

Results of linear models that included supermarket group, SEP and interaction of supermarket group by SEP. * indicates that the interaction of supermarket group by SEP was significant (*p* < 0.05); ^1^ Aldi not included in the linear model for end-of-aisle or checkout displays due to there being insufficient numbers of price promotions in those locations. n/a: not applicable, as Aldi did not have enough observations. Socio-economic position (SEP) based on quintiles of Australian Bureau of Statistics Index of Relative Socio-economic Advantage and Disadvantage. Q1: most disadvantaged. CI: confidence intervals.

**Table 3:** Mean proportion (%) of price-promoted food and beverage display space dedicated to unhealthy products for each in-store island-bin location, by supermarket group and area-level socio-economic position.

	Mean Proportion (%) of Price-Promoted Food and Beverage Display Space Dedicated to Unhealthy Products (95% CIs)			
Supermarket Group, SEP	Island Bins near Checkouts	Island Bins near End of Aisles ^1^	Island Bins near Entrance ^1^	Island Bins Elsewhere in Store
Woolworths	80.9 (71.8, 89.9)	65.9 (54.2, 77.5)	19.4 (8.3, 30.5)	50.2 (40.6, 59.9)
Q1	85.9 (71.3, 100)	67.4 (49.6, 85.3)	15.2 (0, 33.1)	54.7 (39.1, 70.2)
Q2–Q5	77.7 (66.2, 89.3)	64.7 (49.2, 80.1)	22.0 (7.9, 36.1)	47.5 (35.2, 59.7)
Coles	82.4 (73.1, 91.6)	81.3 (70.4, 92.2)	19.3 (8.0, 30.6)	44.3 (34.7, 53.9)
Q1	86.2 (71.6, 100)	77.2 (60.2, 94.1)	14.6 (0, 32.5)	59.3 (43.8, 74.9)
Q2–Q5	79.8 (67.9, 91.7)	84.3 (70.0, 98.6)	22.4 (7.8, 37.0)	34.9 (22.6, 47.2)
Aldi	66.1 (53.7, 78.4)	n/a	n/a	13.7 (1.8, 25.7)
Q1	91.7 (68.6, 100)	n/a	n/a	31.7 (9.8, 53.7)
Q2–Q5	65.6 (48.2, 83.1)	n/a	n/a	6.3 (0, 20.4)
Independents	75.1 (61.2, 89.0)	65.0 (49.6, 80.5)	38.5 (23.3, 53.6)	50.1 (38.5, 61.7)
Q1	75.1 (56.2, 93.9)	57.0 (35.1, 78.8)	19.6 (0, 44.9)	52.8 (36.5, 69.2)
Q2–Q5	59.3 (43.0, 75.6)	73.0 (51.2, 94.9)	48.9 (30.1, 67.8)	47.4 (31.0, 63.8)
Overall	77.8 (72.5, 83.1)	72.2 (65.1, 79.3)	23.5 (16.4, 30.5)	41.3 (36.0, 46.6)
Q1	84.6 (76.2, 93.0) *	68.8 (58.1, 79.5)	15.9 (4.5, 27.2)	52.2 (43.8, 60.6) *
Q2–Q5	73.4 (66.5, 80.2)	74.8 (65.4, 84.3)	28.2 (19.3, 37.2)	34.3 (27.6, 41.1)

Results of linear models that included supermarket group, SEP and interaction of supermarket group by SEP. * Significant difference between Q1 and Q2–Q5 (*p* < 0.05); ^1^ Aldi not included in the linear model for end of aisle or entrance due to there being insufficient numbers of price promotions in those locations. n/a: not applicable, as Aldi did not have enough observations. Socio-economic position (SEP) based on quintiles of Australian Bureau of Statistics Index of Relative Socio-economic Advantage and Disadvantage. Q1: most disadvantaged. CI: confidence intervals.

**Table 4:** Mean magnitude of discount on food and beverage price promotion at prominent in-store supermarket locations.

	End-of-Aisle ^1^				Island Bins			
Supermarket Group, SEP	Mean Magnitude of Discount (95% CIs), %	Mean Magnitude of Discount on Unhealthy Products (95% CIs), %	Mean Magnitude of Discount on Healthy Products (95% CIs), %	Mean Difference in Magnitude of Discount between Healthy and Unhealthy Products (95% CIs), %	Mean Magnitude of Discount (95% CIs), %	Mean Magnitude of Discount on Unhealthy Products (95% CIs), %	Mean Magnitude of Discount on Healthy Products (95% CIs), %	Mean Difference in Magnitude of Discount between Healthy and Unhealthy Products (95% CIs), %
Woolworths	39.4 (37.0, 41.9)	40.3 (37.6, 42.9)	38.4 (35.4, 41.4)	−1.9 (−4.6, 0.8)	34.7 (32.2, 37.3)	34.0 (31.2, 36.9)	35.8 (33.0, 38.7)	1.8 (−0.6, 4.2)
Q1	40.7 (36.8, 44.2)	40.4 (36.1, 44.7)	41.3 (36.6, 46.1)	1.0 (−3.4, 5.3)	34.1 (30.0, 38.3)	32.8 (28.2, 37.4)	36.9 (32.3, 41.5)	4.1 (0.2, 8.0)
Q2−Q5	38.6 (35.6, 41.7)	40.2 (36.8, 43.6)	36.5 (32.7, 40.3)	−3.7 (−7.1, −0.3)	35.1 (31.8, 38.4)	34.8 (31.2, 38.4)	35.2 (31.5, 38.8)	0.4 (−2.7, 3.5)
Coles	38.9 (36.5, 41.3)	38.3 (35.7, 41.0)	40.6 (37.6, 43.5)	2.2 (−0.5, 4.9)	30.1 (27.6, 32.7)	28.5 (25.7, 31.4)	33.8 (30.9, 36.7)	5.2 (2.8, 7.7)
Q1	38.9 (35.0, 42.8)	38.1 (33.8, 42.4)	40.7 (35.9, 45.5)	2.6 (−1.7, 7.0)	29.8 (25.6, 33.9)	27.7 (23.1, 32.3)	34.8 (29.9, 39.6)	6.8 (2.9, 10.8)
Q2−Q5	38.9 (35.8, 42.0)	38.5 (35.1, 41.9)	40.5 (36.7, 44.2)	2.0 (−1.5, 5.4)	30.3 (27.1, 33.6)	29.1 (25.4, 32.7)	33.3 (29.6, 36.9)	4.2 (1.1, 7.3)
Aldi	n/a	n/a	n/a	n/a	31.6 (28.6, 34.7)	32.9 (29.3, 36.5)	31.2 (27.3, 35.1)	−0.6 (−3.5, 2.3)
Q1	n/a	n/a	n/a	n/a	28.5 (23.6, 33.5)	27.8 (22.4, 33.3)	29.1 (22.6, 35.7)	1.3 (−3.3, 6.0)
Q2−Q5	n/a	n/a	n/a	n/a	33.6 (29.7, 37.6)	36.8 (32.0, 41.7)	32.3 (27.5, 37.2)	−1.8 (−5.5, 1.9)
Independents	30.4 (27.7, 33.2)	31.2 (28.2, 34.2)	33.2 (29.4, 36.9)	0.3 (−2.8, 3.3)	29.0 (26.1, 31.9)	30.8 (27.6, 34.1)	27.6 (23.8, 31.4)	−2.9 (−5.7, −0.1)
Q1	33.1 (29.0, 37.3)	32.9 (28.4, 37.4)	33.4 (27.7, 39.2)	0 (−4.6, 4.5)	30.9 (26.6, 35.3)	32.8 (27.9, 37.6)	27.0 (21.8, 32.2)	−3.2 (−7.3, 0.9)
Q2−Q5	28.2 (24.5, 31.9)	29.8 (25.7, 33.9)	32.9 (28.0, 38.0)	0.5 (−3.6, 4.6)	27.4 (23.5, 31.4)	29.3 (23.0, 35.5)	28.3 (22.8, 33.8)	−2.6 (−6.4, 1.1)
Overall	36.7 (35.3, 38.2)	37.1 (35.5, 38.7)	38.0 (36.1, 39.8)	0.2 (−1.4, 1.8)	31.5 (30.1, 32.9)	31.5 (24.9, 33.6)	32.8 (31.2, 34.5)	1.3 (0, 2.6)
Q1	37.7 (35.4, 40.0)	37.3 (34.7, 39.8)	39.1 (36.2, 42.0)	1.2 (−1.3, 3.8)	31.0 (28.9, 33.2)	30.4 (28.0, 32.8)	32.6 (30.0, 35.2)	2.5 (0.4, 4.6)
Q2−Q5	36.1 (34.2, 38.0)	36.9 (34.8, 39.0)	37.3 (34.9, 39.6)	−0.5 (−2.6, 1.7)	31.8 (30.0, 33.6)	32.2 (30.2, 34.2)	33.0 (30.9, 35.1)	0.5 (−1.2, 2.1)

Results of linear models that included supermarket group, SEP and interaction of supermarket group by SEP. ^1^ Aldi not included in the linear model for end-of-aisle displays due to there being insufficient numbers of price promotions in those locations. n/a: not applicable, as Aldi did not have enough observations. Socio-economic position (SEP) based on quintiles of Australian Bureau of Statistics Index of Relative Socio-economic Advantage and Disadvantage. Q1 = most disadvantaged. CI: confidence intervals.

## Supplementary Materials

The following are available online at https://www.mdpi.com/1660-4601/17/23/9026/s1. File S1: Monitoring Availability, Placement and Promotion—Supermarkets (MAPP-S) Audit Tool (developed for this study).
