# Supplementary material for: The Healthiness of Food and Beverages on Price Promotion at Promotional Displays: A Cross-Sectional Audit of Australian Supermarkets
Source: Int J Environ Res Public Health. 2020 Dec 3;17(23):9026. doi: 10.3390/ijerph17239026 (PMC7729449; doi:10.3390/ijerph17239026)
Supplement: Supplementary file 1 [file ijerph-17-09026-s001.pdf]

File S1. Monitoring Availability, Placement and Promotion – Supermarkets (MAPP-S) Audit Tool (developed for this study).

1. STORE DETAILS

1a. Supermarket chain (circle one): Aldi Coles Woolworths Independent (specify store name): ______________________________

1b. Supermarket address: ____

1c. Auditor name: _____________________________ 1d. Date of audit:__________________ 1e. Start time:__________ 1f. Finish time:___________

1g. Store manager notified of data collection (circle one)? Yes / No

1h. Name of manager providing consent (Independents only): ____

1i. Total supermarket floor perimeter[i]: ____________________ (metres (m))

2. SHELF-SPACE OF SELECTED HEALTHY AND UNHEALTHY FOOD

2a. Frozen products

- Frozen aisle/freezer containing **frozen fruit** shelf length _____________ x height[ii]_____________(m)
- Frozen aisle/freezer containing **frozen vegetables**[iii] shelf length _____________ x height[ii]_____________(m)
- Frozen aisle/freezer containing **ice cream**[iv] shelf length _____________ x height[ii]_____________(m)

2b. Fresh fruit and vegetables

- Refrigerated /chilled section containing **fresh fruit and vegetables**[v]= shelf length _____________ x height[ii]_____________(m)

---

[i] Starting at the entrance of the store, measure total floor space, using a measuring wheel, around the perimeter of the store only (inside the store and on the inside of the checkouts, where feasible to measure) – do not include aisles

[ii] Height is measured from the bottom of the lower shelf to the top of the upper shelf, using a tape measure. Shelf length measured using a measuring wheel.

[iii] **Excluding frozen potato** products and **ready to eat meals**

[iv] **Icecream** and **ice lollies included. Frozen desserts excluded**. Free-standing frozen display containing single serve ice-creams to be included as an Island Bin.

[i] Including packaged herbs, chilli and garlic/ginger/herb puree display

- Display bins for **fresh fruit and vegetables**[i]:

Bin no.	Length (m)	Width (m)
1		
2		
3		
4		
5		
6		

Bin no.	Length (m)	Width (m)
7		
8		
9		
10		
11		
12		

Free fruit available for children: Y / N

2c. Selected discretionary products

- Chocolate and confectionery[ii]: shelf length ____________ x height[iii]____________(m)
- Chips, pretzels, popcorn (incl. multipacks[iv]): shelf length ____________ x height[2]____________(m) *Check other aisles eg. Health Food aisle.
- Sweet biscuits: shelf length ____________ x height[2]____________(m)
- Soft drinks and energy drinks[v]: length __________ x height[ii]__________(m) Sports drinks and iced tea length __________ x height[ii]__________(m)

---

[i] Fruit and veg display include free standing and permanent displays, in the F&V section of the store only. **Potatoes, onions, ginger and garlic are included**. **Nuts of all kinds are excluded**.
[ii] Including gums and mints
[iii] Height is measured from the bottom of the lower shelf to the top of the upper shelf, using a tape measure. Shelf length measured using a measuring wheel.
[iv] **Pretzels** and **popcorn** are **included.** Check other aisles (e.g. health food section) for crisps
[v] **Sugar-sweetened** and **artificially-sweetened** varieties are **included**

3. END-OF-AISLE (EOA) DISPLAYS *(See Addendum A for examples of EOA displays)*

3a. EOA - FRONT *(located directly opposite the front of the store / checkouts)*

Aisle no.	**Left** wing of **EOA - Front** (record broad product type, including non-food)	Estimated % of space the product takes up in display	Price promotion type (1-9) *See price promotions (Addendum B)*	**Centre** (main) section of **EOA – Front** (record broad product type, including non-food)	Estimated % of space the product takes up in display	Price promotion type (1-9) *See price promotions (Addendum B)*	**Right** wing of **EOA - Front** (record broad product type, including non-food)	Estimate d % of space the product takes up in display	Price promotion type (1-9) *See price promotions (Addendum B)*

3b. EOA - OTHER *(EOA located anyway except opposite front of store, including rear of store or in the middle of store where aisles are separated)*

Aisle no.	**Left** wing of **EOA – Other** (record broad product type, including non-food)	Estimated % of space the product takes up in display	Price promotion type (1-9) *See price promotions (Addendum B)*	**Centre** (main) section of **EOA - Other** (record broad product type, including non-food)	Estimated % of space the product takes up in display	Price promotion type (1-9) *See price promotions (Addendum B)*	**Right** wing of **EOA - Other** (record broad product type, including non-food)	Estimated % of space the product takes up in display	Price promotion type (1-9) *See price promotions (Addendum B)*

4. ISLAND BINS - FOOD AND BEVERAGES ONLY [i] *(See Addendum A for examples of island bins)*

**Island bins - food and beverages only**					
**Bin no.**	**Bin Size**[ii]<br>S=Small (<1m^2)<br>M=Medium 1-1.5m^2)<br>L= Large (1.5-2m^2)<br>XL=Extra-large (>2m^2)	**Product displayed (record broad product type)**	**Estimated % of space the product takes up in display**	**Location of island bin (specify no.)**<br>1. Near entrance<br>2. Near checkout<br>3. Near self-checkout<br>4. Near end of aisle<br>5. Elsewhere	Price promotion type (1-9)<br>*See price promotions (Addendum B))*

[i] Island bins are defined as temporary displays separate from the main aisles or permanent displays. They include items that are elsewhere in the store – if the bin is the only location in the store for that product, then this is a permanent display and not an island bin, except for single serve items (eg. ice-creams and soft drink).

[ii] Measure height (H) and width (W) of island bins using measuring tape or measuring wheel and calculate area (HxW) in square metres.

5. CHECKOUTS *(See Addendum A for examples of checkout displays)*

5a. Staff-assisted checkouts

Check-out no.	End checkout display[i] (record broad product type, including non-food)	Estimated % of space the product takes up in display	Is there a promotional display?[ii] T = Yes, temporary P = Yes, permanent N = No	Side checkout display[iii] (record broad product type, including non-food)	Estimated % of space the product takes up in display	Is this product on price promotion? T = Yes, temporary P = Yes, permanent N = No	Was this checkout promoted as 'junk food/ confectionery-free'? (Y/N)

[i] End checkouts are located at the entrance of the checkout, facing the inside of the store. Displays that are located between two checkouts are recorded twice (at both checkouts)
[ii] Temporary promotions i.e. discounts valid up to a week. Permanent promotions i.e. promotion of non-temporary low price (e.g. "down down"). Refer to promotional display card
[iii] Side checkouts run alongside the checkout, usually above the conveyer belt.

**5b. Self- checkouts**			
Checkout no.	Checkout display[i] (record broad product type, including non-food)	Estimated % of space the product takes up in display	Is there a promotional display? T = Yes, temporary P = Yes, permanent N = No

[i] Displays that are located between two checkouts are recorded twice (at both checkouts)

**Self-checkouts** (continued)			
Checkout no.	Checkout display<br>(record broad product type, including non-food)	Estimated % of space the product takes up in display	Is there a promotional display?<br>T = Yes, temporary<br>P = Yes, permanent<br>N = No

6. GENERAL OBSERVATIONS (eg. old format store, renovation underway, Mother's Day specials present)

ADDENDUM A – EXAMPLES OF DISPLAYS

END-OF-AISLE DISPLAY

Left wing

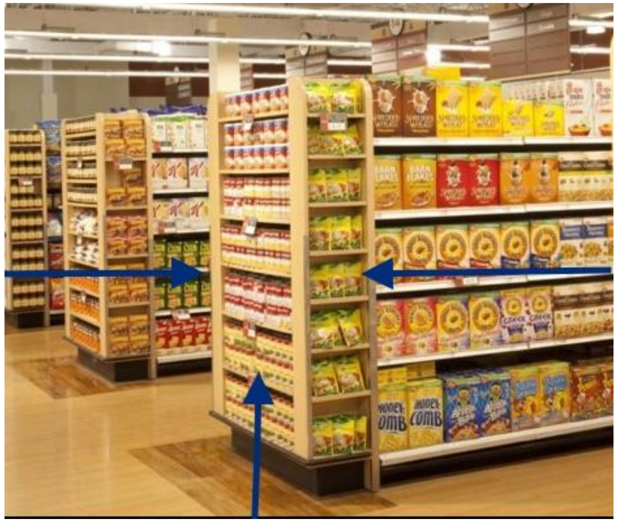

Right wing

Centre (main) section

ISLAND BINS

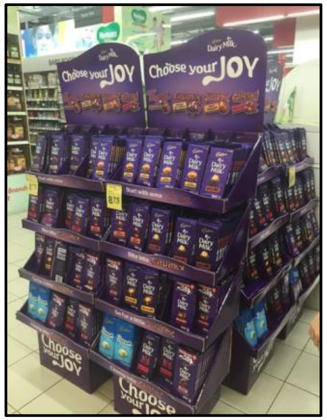

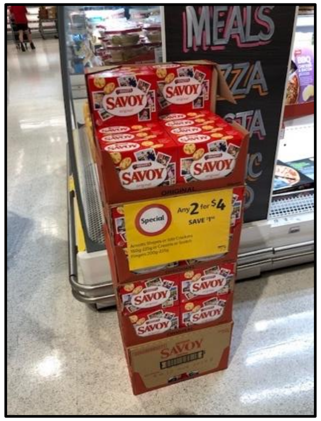

STAFF-ASSISTED CHECKOUT DISPLAYS

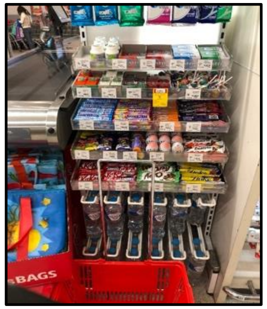

End display

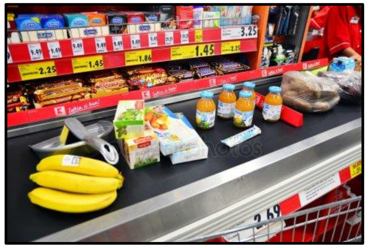

Side display

SELF-CHECKOUT DISPLAYS

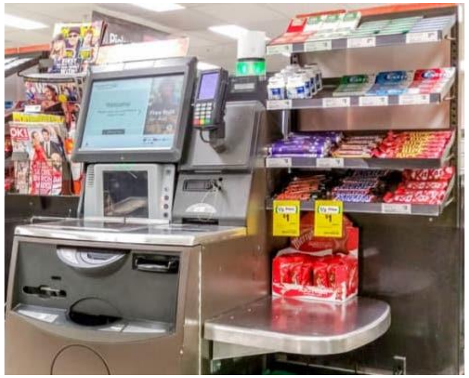

ADDENDUM B - PRICE PROMOTION CODES

1. No price promotion

Temporary

2. 25% off
3. 50% off/half price
4. Other % discount quoted (specify discount eg. 33% off, if not quoted, record RRP and sale price)
5. Multibuys (specify e.g. 2 for 1)
6. Other price discount (specify e.g. buy 2 [RRP $1] get 50c off third one; introductory offer [$5.99] will be $7.99])
7. "Fresh Specials" or similar (fruit/veg with no RRP)

Permanent

8. "price dropped", "down down", or similar
9. "Everyday low price" or similar

NOTES:

Temporary price promotions: Promotions that are valid for up to 1 week;

Permanent price promotions: promotions that are valid or have been offered for more than 1 week.

Note: items labelled "price dropped", "specials", or similar may fall into temporary or permanent categories.

Products reduced to clear/on sale based on expiry date are **excluded.**

Products that are on offer as part of an introductory offer are **included**
